# The Role of Telomerase in Cellular Stress Response—Therapeutic Potential of Telomerase Inhibitors

**DOI:** 10.3390/biomedicines14102171

**Published:** 2026-09-25

**Authors:** Sandra Pawlak, Magdalena Kostrzewa, Natalia Lisiak, Blazej Rubis

**Affiliations:** 1 Doctoral School, Poznan University of Medical Sciences, 60-812 Poznan, Poland; sandra.pawlak@student.ump.edu.pl; 2 Department of Clinical Chemistry and Molecular Diagnostics, Poznan University of Medical Sciences, 60-806 Poznan, Poland; 87114@student.ump.edu.pl (M.K.); nlisiak@ump.edu.pl (N.L.); 3 Burnett School of Biomedical Sciences, College of Medicine, University of Central Florida, Orlando, FL 32827, USA

**Keywords:** telomerase, hTERT, telomeres, non-canonical functions, oxidative stress, cellular stress response, cancer therapy resistance, treatment sensitization, telomerase inhibitors, G-quadruplexes

## Abstract

Cancer remains one of the most complex diseases and a major global problem. Current therapeutic strategies often fail because of severe side effects and insufficient selectivity. Thus, there is a need to identify novel, specific targets and personalized therapeutic approaches. Telomerase may be such a target. Although it has been studied primarily for its role in telomere maintenance and cellular immortality, its broader roles in cancer biology remain poorly understood. Many approaches targeting telomerase expression and activity focus on its primary function of restoring telomeres. However, studies show that the catalytic subunit of telomerase (TERT) shows additional functions that are independent of telomere restoration. These non-canonical functions support cancer cell survival, increased tolerance to cytotoxic stress, and enable tumor cells to adapt to adverse microenvironmental conditions. The mechanism by which non-canonical TERT functions in cellular stress responses (also due to therapeutic agents’ effects) remains unclear, and its therapeutic relevance is not yet fully understood. This review discusses the role of non-canonical telomerase functions in cancer cell stress responses and highlights the therapeutic potential of TERT/telomerase inhibition.

## 1. Introduction

Cancer remains a major global health problem. Recent global estimates reported close to 20 million new cancer cases and 9.7 million cancer-related deaths worldwide [1]. Several common cancer types have high cure probabilities when detected early and treated effectively [2]. Early diagnosis and personalized therapy require the identification of molecular signatures that define tumor biology, predict treatment response, and reveal therapeutic vulnerabilities [3].

Hallmarks of cancer include uncontrolled proliferation, resistance to cell death, metastatic spread, and survival under adverse conditions [4]. Cancer cells are characterized by the activation of survival pathways that maintain long-term viability, allowing them to bypass the replication limits and cellular aging [5]. Telomerase is one of the key factors sustaining the long-term proliferative capacity of cancer cells [6].

The preclinical studies discussed below were selected because they directly address stress responses involving telomerase or hTERT, as well as telomerase-targeting strategies in the context of cancer therapy. The strength of the available evidence varies: some mechanisms have been supported across different experimental models, whereas others are based on individual cell-line studies or have not yet been independently validated in vivo. In addition, the available literature may be influenced by publication bias, with positive mechanistic findings being reported more frequently than negative or inconclusive results. Therefore, model-specific findings are interpreted cautiously, particularly for non-canonical hTERT functions, for which apparently conflicting effects have been reported. In this review, resistance, stress adaptation, sensitization, potentiation, and synergy are treated as distinct biological and experimental outcomes, rather than interchangeable terms (Box 1).

Box 1 **Terminology used to describe hTERT-dependent treatment responses.**

Resistance—a reduction in the efficacy of a defined anticancer treatment associated with telomerase/hTERT activity or expression.

Stress adaptation—an increased capacity to tolerate adverse conditions, such as oxidative, metabolic, hypoxic, or genotoxic stress, without necessarily demonstrating resistance to a defined treatment.

Sensitization—an increased response to a treatment following genetic or pharmacological suppression of telomerase/hTERT. Sensitization does not by itself demonstrate a synergistic interaction.

Potentiation—an increased effect of one intervention in the presence of another. This term is used descriptively when a formal interaction analysis has not been performed.

Synergy—a combination effect that exceeds the effect expected under a predefined quantitative interaction model, such as Chou–Talalay combination index, Bliss independence, or Loewe additivity.

## 2. Telomeres and Telomerase

### 2.1. Telomeres

Telomeres are nucleoprotein complexes located at the ends of eukaryotic chromosomes. In mammals, telomeric DNA consists of tandem 5′-(TTAGGG)_n_-3′ repeats that can span from a few to several tens of kilobases, depending on cell type, age, and species [7]. The shelterin complex, composed of six proteins (TRF1, TRF2, POT1, TIN2, TPP1, and RAP1), binds to telomeric repeats, protecting chromosome ends from degradation, from joining, being recognized as a DNA break, and regulating telomerase access (Table 1) [8,9].

While shelterin protects chromosome ends from being recognized as DNA breaks, this protective masking can also limit the recruitment of DNA repair factors, contributing to the persistence of telomeric lesions under oxidative stress [11,12].

At the telomere end, the two DNA strands differ in length. The G-rich strand extends beyond the C-rich strand. This asymmetry creates a single-stranded 3′ overhang [13]. The 3′ G-rich overhang folds back toward the double-stranded telomeric region and invades the duplex, pairing with the complementary C-rich strand and displacing the original G-rich strand. This strand invasion forms a displacement loop (D-loop) at the base of a larger telomeric loop (T-loop) [14]. These looped structures hide chromosome ends from DNA damage sensors and repair systems [11,15].

### 2.2. Telomerase

Telomerase is a ribonucleoprotein (RNP) enzyme that extends the 3′ ends of linear chromosomes and restores telomeric repeats lost during each cell division [16]. In humans, its activity relies on two core components: the catalytic protein subunit, human telomerase reverse transcriptase (hTERT), and the human telomerase RNA component (hTERC or hTR), which serves as the RNA template. Together, hTERT and hTERC are sufficient to sustain telomerase activity in vitro [17,18]. In vivo, the human telomerase complex includes additional proteins bound to hTERC, such as dyskerin (DKC1), NOP10 ribonucleoprotein (NOP10), NHP2 ribonucleoprotein (NHP2), and glycine-arginine-rich protein 1 (GAR1) [19,20]. These auxiliary proteins stabilize hTERC, support proper folding of the telomerase RNP, and promote assembly of the active holoenzyme at chromosome ends [21,22].

Telomerase activity is usually low or undetectable in most normal somatic cells, whereas it is detected in most human cancers (up to 90%), where it supports telomere maintenance during sustained proliferation [6,7,23].

### 2.3. Telomere Maintenance Mechanisms and TERT Regulation in Cancer

Telomerase-dependent telomere maintenance is not universal in cancer. Some tumors maintain their telomeres through alternative lengthening of telomeres (ALT), a telomerase-independent mechanism based on recombination-mediated telomere synthesis [24]. ALT is often associated with alterations in the chromatin regulators ATRX chromatin remodeler (ATRX) and death domain associated protein (DAXX), although this relationship differs between tumor types [25]. Accordingly, ALT-positive tumors may be less responsive to therapies that rely on inhibition of canonical telomerase activity.

TERT expression alone is not sufficient to induce malignant transformation. Ectopic hTERT expression can extend the proliferative lifespan of normal human cells and, in some models, lead to immortalization without malignant transformation [26]. TERT expression can also occur in selected normal proliferative cell populations and early premalignant lesions [27]. In cancer, TERT can be activated through several mechanisms, including promoter mutations, structural rearrangements, and epigenetic or transcriptional deregulation [28]. TERT promoter mutations create de novo binding motifs for ETS-family transcription factors [29]. GA-binding protein (GABP), a member of the ETS family, can bind these mutant promoters and activate TERT transcription [30]. Structural rearrangements can reposition strong enhancer elements near the TERT locus, as shown in high-risk neuroblastoma [31]. Altogether, the activation of TERT expression in cancer cells is primarily driven by genetic mutations, structural variations, and epigenetic changes that override the strict repression normally seen in somatic cells [32]. Epigenetic regulation of TERT is discussed in more detail in Section 4.3.

Alternative splicing provides an additional post-transcriptional level of TERT regulation. Long-read sequencing of selected telomerase-positive human cell models recently identified 45 TERT mRNA splice variants, including 13 previously described and 32 novel transcripts. Current data do not show that all of these transcripts produce stable or functional protein isoforms [33]. Some TERT splice variants lack catalytic activity but retain biological functions. For example, overexpression of the β-deletion variant inhibited endogenous telomerase activity while reducing cisplatin-induced apoptosis in breast cancer cells [34]. The recently described TERT Δ2–4 variant also increased clonogenic survival and resistance to cisplatin-induced cell death in lung cancer cells without increasing telomerase activity or measurably altering telomere length [33]. TERT alternative splicing may contribute to treatment response independently of canonical telomere maintenance. Direct evidence linking specific TERT splice variants to radioresistance remains limited [35].

The timing of TERT activation depends on tumor context. In melanoma, TERT promoter mutations can occur before stable telomere maintenance is established. Telomeres may continue to shorten during this early phase, while limited telomerase activity preferentially maintains the shortest telomeres and extends cellular lifespan. Further increases in telomerase activity can later support more stable telomere maintenance and sustained proliferation [36]. TERT activation can precede the establishment of a stable telomerase-dependent phenotype.

## 3. Role of Telomerase and Telomeres in Cellular Stress Response Mediated by Mitochondria

hTERT may influence cellular stress responses through mitochondrial mechanisms that are independent of direct telomere elongation.

### 3.1. Key Mechanisms of the Mitochondrial Response to Oxidative Stress

Mitochondria are central regulators of cellular stress responses, integrating metabolic signaling, redox homeostasis, and apoptotic pathways [37]. As both sources and targets of reactive oxygen species (ROS), mitochondria occupy a pivotal position in oxidative stress regulation. They generate ROS as byproducts of oxidative phosphorylation [38] and dynamically respond to changes in redox status, thereby influencing cell fate decisions [39,40]. Mitochondrial ROS levels are limited by antioxidant enzymes, including manganese superoxide dismutase (SOD2), glutathione peroxidases, and the mitochondrial thioredoxin system, which detoxify superoxide and hydrogen peroxide (H_2_O_2_) generated by the respiratory chain [41]. In cancer cells, mitochondrial redox regulation is frequently altered, supporting proliferation and adaptation to persistent oxidative pressure [42]. Several integrated molecular mechanisms govern the mitochondrial response to cellular stress.

### 3.2. Differences in Redox Response Between Normal and Cancer Cells

Reactive oxygen species (ROS) were initially treated mainly as harmful byproducts of metabolism. This view is incomplete. At physiological levels, ROS are required for normal organismal function because they enable redox signaling and support homeostatic control [43,44]. Throughout evolution, organisms developed regulatory systems that maintain redox homeostasis [45,46]. When these controls weaken, oxidative stress develops and is prevalent in cancer [47]. In malignant cells, the defining feature is a shift toward a chronically perturbed redox state. Many tumors persist under sustained oxidative pressure [48]. This baseline condition determines oncogenic potential, therapeutic selectivity, and the emergence of resistance [49]. One of the critical mechanisms that may enable cancer cell survival under redox stress is the ability of hTERT to translocate or localize to mitochondria [50,51,52]. The exact protective role of the telomeric catalytic subunit is not yet known, but there are many reports demonstrating its involvement in redox adaptation, mtDNA maintenance, and apoptosis control.

### 3.3. Translocation and Mitochondrial Localization of TERT

Oxidative stress may affect whole-cell metabolism including telomere stability. Interestingly, stress was also associated with the translocation of the telomerase catalytic subunit from the nucleus to the cytoplasm, suggesting that a fraction of this protein localizes to mitochondria [50,51,52]. This links telomerase/hTERT to mitochondrial homeostasis and cellular stress responses that do not depend directly on its canonical role in telomere maintenance [53,54]. Recent studies also suggest that mitochondrial localization of TERT is not restricted to oxidative stress conditions, since TERT has also been detected in mitochondria under basal conditions [51,55]. However, the mechanism of TERT redistribution and its consequences for mitochondria remain unresolved. Here, nuclear export and mitochondrial import are treated as related events, but not as evidence that the same mature hTERT molecule rapidly moves from the nucleus to mitochondria through a single continuous pathway.

Current evidence suggests that two separate transport processes may underlie this phenomenon. The first is nuclear export. Under oxidative stress, hTERT undergoes Src-dependent phosphorylation at Tyr707, promoting its export from the nucleus through a CRM1-dependent pathway [50]. The second is mitochondrial import. TERT contains an N-terminal mitochondrial targeting sequence (MTS), enters mitochondria by interacting with the translocases of the outer mitochondrial membrane 20 and 40 (TOM20/40) and the translocase of the inner mitochondrial membrane 23 (TIM23) complex, and accumulates in the matrix [51,52]. The classical model assumes such translocation under stress, but recent work did not detect rapid transfer of mature nuclear TERT-SNAP to mitochondria within three hours of H_2_O_2_ treatment [56]. This raises the possibility that at least part of the mitochondrial pool may derive from the direct import of newly synthesized cytosolic hTERT. These results should be interpreted with caution because the experimental approach had important limitations. The C-terminal SNAP fusion may also have affected TERT redistribution, because the tag was attached close to the FKT sequence at position 1127, which is involved in the regulation of TERT localization [56,57]. In addition, H_2_O_2_ increased nonspecific cytoplasmic background signal, which may have made TERT redistribution difficult to detect [56]. Thus, the TERT-SNAP study challenges the idea of a single continuous nucleus-to-mitochondrial trafficking route, but it does not exclude it. In the context of non-canonical TERT functions, the intracellular transport of TERT and its translocation from the nucleus to the mitochondria in response to oxidative stress remain a subject of debate in cancer cell biology.

### 3.4. Functional Implications of Mitochondrial TERT

The biological role of mitochondrial TERT remains controversial. In several models, TERT is associated with a protective phenotype characterized by lower ROS levels, preservation of mitochondrial membrane potential, and improved respiratory chain activity [58,59,60]. TERT has also been reported to physically associate with mtDNA. Initial studies identified selective binding to regions encoding NADH dehydrogenase subunits 1 and 2 (ND1 and ND2) of mitochondrial complex I [51]. However, more recent evidence indicates that TERT associates with multiple regions of the mitochondrial genome, including those encoding NADH: ubiquinone oxidoreductase subunits 3 and 5 (ND3 and ND5), and cytochrome c oxidase subunit 1 (COX1), suggesting that this interaction may not be strictly sequence-specific [55,61]. These findings raise the possibility that TERT may influence mtDNA maintenance more broadly than acting through a single specific genomic locus. In this view, mitochondrial TERT may help maintain organellar integrity and support cell survival during chronic or moderate oxidative stress.

By contrast, two studies from the same research group reported a damaging effect of mitochondrial hTERT under oxidative stress. The earlier study found that mitochondrial hTERT exacerbated H_2_O_2_-mediated mtDNA damage [52], whereas the subsequent study linked mitochondrial hTERT localization to increased H_2_O_2_-induced mtDNA damage and apoptosis [62]. This effect may be related to increased availability of redox-active divalent metals, which can promote hydroxyl radical (^•^OH) formation near mtDNA [52]. In addition, impaired TERT shuttling caused by nuclear retention was associated with increased telomeric and non-telomeric DNA damage, reduced colony formation, increased mtDNA damage, and higher sensitivity to genotoxic stress [63]. These opposing findings suggest that the mitochondrial effect of TERT is context-dependent [60,64].

However, the conflicting reports regarding the role of mitochondrial TERT (mtTERT) should not be viewed as mutually exclusive, but rather as a reflection of its dualistic, context-dependent nature. mtTERT functions as a metabolic “decision sensor” in cancer cells—exacerbating organelle dysfunction and accelerating apoptosis under acute stress, while optimizing respiration and promoting survival during chronic adaptation. Resolving this paradox requires a critical shift away from artifactual overexpression systems toward the investigation of endogenous TERT within the specific metabolic landscape of individual tumors.

Recent studies also suggest that mitochondrial TERT plays a role beyond redox homeostasis, encompassing mitochondrial dynamics and metabolic adaptation. TERT has been linked to mitochondrial fission, mitophagy, and organelle turnover [55,59]. In papillary thyroid carcinoma models, these changes were associated with increased AKT phosphorylation at Ser473 and higher lactate production, suggesting a shift toward aerobic glycolysis, consistent with the Warburg effect [59].

An additional issue is whether mitochondrial effects attributed to TERT reflect the activity of the complete telomerase holoenzyme or a non-canonical function of the TERT protein itself. This distinction is particularly relevant because TERT and TERC appear to occupy different submitochondrial compartments. TERT is mainly detected in the mitochondrial matrix, whereas the RNA component TERC remains largely restricted to the intermembrane space (IMS) [55]. In this compartment, TERC is processed by the ribonuclease RNASET2 into a shortened 53-nucleotide form, TERC-53, which is then exported to the cytosol and acts as a retrograde signal involved in the regulation of cellular senescence [65,66]. This spatial separation of the two core telomerase components suggests that they may not function together within the same mitochondrial compartment. Therefore, at least some effects attributed to mitochondrial telomerase may instead reflect functions of TERT itself, independent of TERC and the telomerase holoenzyme. This would support interpreting these mitochondrial effects as a non-canonical function of TERT rather than canonical telomerase activity. Whether TERT and TERC transiently form a functional complex within mitochondria remains unresolved. Figure 1 summarizes how oxidative telomere damage, mitochondrial dysfunction, and reported hTERT redistribution may converge in hTERT-dependent stress modulation.

### 3.5. Possible Explanations for Divergent Mitochondrial TERT Effects

The divergent effects of mitochondrial TERT may reflect differences in stress conditions rather than mutually exclusive functions of the protein. One possible explanation is the intensity and duration of oxidative stress. Under moderate or transient stress, mitochondrial TERT may support mitochondrial function and limit oxidative damage, whereas stronger or prolonged stress may exceed this protective capacity. The basal redox state of the cell may also shift this response. Cancer cells differ in mitochondrial ROS production and antioxidant capacity, which may influence whether mitochondrial TERT supports adaptation or is associated with increased susceptibility to stress [60,64]. These possibilities could be tested by varying stress intensity and duration within the same cellular model and by comparing cells with different basal redox states under matched stress conditions. Changes in mitochondrial ROS, mtDNA damage, and cell survival could then be compared with TERT localization.

The regulatory state of TERT may provide another explanation. Oxidative stress promotes Src-dependent phosphorylation of TERT at Tyr707 and its nuclear export [50,51], but it remains unclear whether this or other post-translational modifications also influence TERT after mitochondrial import. Differences in cell type and experimental conditions may further contribute to the reported discrepancies. To test whether TERT regulation itself contributes to these differences, TERT phosphorylation, nuclear export, or mitochondrial targeting could be altered while keeping the stress conditions constant. A change in the mitochondrial response under these conditions would indicate that the regulatory state of TERT contributes to the divergent phenotypes.

## 4. Telomerase Inhibitors

Many cancer cells depend on telomerase for sustained proliferation and replicative immortality [4,6,23]. This makes telomerase a relevant target in cancer therapy [7]. Especially since targeting telomerase triggers some additional effects that are associated with its non-canonical roles. Telomerase inhibitors can be broadly classified into direct and indirect agents, depending on the level at which they interfere with telomerase function. Direct inhibitors act on the telomerase complex itself, whereas indirect inhibitors interfere with telomerase function by altering telomere structure, limiting telomerase access to DNA, or reducing hTERT expression [7,67].

These inhibitor classes may affect the cellular stress response through different mechanisms. Catalytic inhibition primarily impairs long-term telomere maintenance, whereas structural approaches can induce early telomere dysfunction, and transcriptional approaches can reduce hTERT-dependent stress responses. Because hTERT contributes to oxidative stress responses, mitochondrial homeostasis, and apoptosis control, telomerase targeting may affect both telomere maintenance and early stress adaptation [51,58,60,68]. Table 2 summarizes the main inhibitor classes.

### 4.1. Direct Inhibition of Telomerase Activity

Direct enzymatic inhibitors reduce catalytic activity without primarily changing telomeric DNA structure or suppressing hTERT transcription. This separates catalytic inhibition from indirect effects on telomeric DNA or hTERT expression.

BIBR1532 is one of the best-characterized small-molecule inhibitors of human telomerase. In enzymatic assays, BIBR1532 inhibited telomerase processivity with a half maximal inhibitory concentration (IC_50_) of 93 nM [69]. It also inhibited recombinant, affinity-purified telomerase, supporting a direct effect on telomerase catalytic activity [69]. Kinetic analysis using native and recombinant human telomerase showed mixed-type non-competitive inhibition and reduced processivity. This means that telomerase added fewer telomeric repeats before dissociating from the DNA substrate [70]. Recent cryo-electron microscopy structures of human telomerase located the BIBR1532-binding pocket between the finger and palm regions of the hTERT reverse transcriptase domain. BIBR1532 acts allosterically and restricts the conformational changes required for template–DNA duplex repositioning during telomere repeat synthesis [71].

Direct catalytic inhibition usually weakens telomere maintenance over time, and cytotoxic effects appear only after telomeres become dysfunctional. Long-term exposure to BIBR1532 reduced telomerase-dependent telomere maintenance and led to progressive telomere shortening in telomerase-positive cancer cells [69]. Proliferation arrest and hallmarks of senescence appeared only later, when telomeres became critically short or dysfunctional [69]. MST-312 followed a similar pattern at non-toxic concentrations, producing gradual telomere shortening, slower proliferation, and senescence-associated β-galactosidase (SA-β-Gal) activity [72]. Direct inhibition reduces telomere restoration after each cell division, which causes telomere loss, but at different rates for different chromosomes. Eventually, the shortest telomeres become the proliferation-limiting factor [85].

Thus, direct telomerase inhibition does not usually cause immediate telomeric damage. Instead, it gradually weakens the ability of telomerase-positive cells to sustain long-term proliferation. Once telomeres are critically short or dysfunctional, cells activate a telomere-associated DNA damage response. In cells with functional p53, this leads to p53 activation, p21 induction, and cell-cycle arrest associated with senescence or apoptosis [86,87]. Direct catalytic inhibition reveals telomerase dependence by shifting chronic proliferative stress toward telomere dysfunction.

A recent study in B-cell lymphoma models suggests that this picture is not always purely length-dependent. In that study, short-term TERT inhibition impaired proliferation before measurable telomere shortening and was associated with lower NF-κB p65, reduced MYC, and p21-mediated cell cycle arrest [88]. These data do not replace the canonical telomere-shortening model. They do suggest that acute responses to TERT inhibition can also expose non-canonical hTERT survival functions in selected cellular contexts. Early responses to TERT inhibition may therefore reflect disruption of non-canonical hTERT functions, whereas delayed effects are more likely to result from progressive telomere dysfunction.

A key limitation of MST-312 is its dose-dependent lack of selectivity. Later work showed that higher concentrations also inhibit DNA topoisomerase II and induce rapid telomeric and non-telomeric DNA damage [89]. Rapid cytotoxicity after MST-312 treatment, therefore, cannot be interpreted as a purely on-target effect of telomerase inhibition. This may partly explain why direct inhibitors often show stronger effects in combination therapies than as monotherapies. BIBR1532 enhanced radiosensitivity in non-small cell lung cancer (NSCLC) models [90], and a more recent study also reported increased radiation response after BIBR1532 treatment [91]. Direct small-molecule inhibitors such as BIBR1532 and MST-312 nevertheless remain preclinical.

BIBR1532 shows why direct small-molecule telomerase inhibitors have been difficult to translate. Although it inhibits purified telomerase at low concentrations, its canonical cellular effect depends on prolonged exposure, because telomere shortening develops over many cell divisions [69,70]. At higher concentrations, BIBR1532 can produce rapid cytotoxic effects that are difficult to separate from delayed telomerase inhibition [92]. In a human induced pluripotent stem cell (iPSC) model, concentrations compatible with continued culture did not reduce TERT expression, whereas concentrations used in earlier studies were cytotoxic [93]. This makes it difficult to define a clinically useful exposure range, especially because human pharmacokinetic and dose-limiting toxicity data are still lacking.

By contrast, imetelstat belongs to a different class and acts through an oligonucleotide-based mechanism, as discussed below. Recent structural work identified telomerase holoenzyme assembly as a potential target of direct inhibition. Human telomerase can form a dimeric complex, and disruption of the protomer interface impaired RNP assembly, telomerase activity, and telomere maintenance in cells [94].

### 4.2. G-Quadruplex Stabilization as Telomerase Substrate Blockade

Beyond canonical duplex DNA and higher-order telomeric conformations such as T-loops and D-loops, telomeric sequences can form non-canonical G-quadruplex (G4) structures [10]. G4s comprise stacked G-tetrads formed by Hoogsteen hydrogen bonding between guanine bases and stabilized by π–π interactions and monovalent cations [95,96]. Similarly, in the human genome, G4 structures can also be found in the promoter regions of cancer-related genes, including *hTERT*, *c-MYC*, *BCL2*, *KRAS*, *VEGF*, and *HIF1α* [97,98]. These structures can regulate gene expression and have attracted interest as targets for reducing cancer-relevant transcription [99,100]. Ligands targeting G4s at telomeric ends and within the *TERT* promoter could stabilize these structures, potentially inhibiting *TERT* (and other genes, including oncogenes) expression and limiting telomere extension [75]. This has motivated the development of small-molecule G4-binding ligands as potential anticancer therapeutics [101,102,103].

G4 stabilizers inhibit telomerase indirectly by stabilizing G4 structures formed by the G-rich single-stranded 3′ telomeric overhang. This folded substrate limits telomerase access to chromosome ends and reduces telomere extension [76,104].

Early work with RHPS4, a pentacyclic acridine G4 ligand, provided experimental support for this mechanism. RHPS4 inhibited telomerase access to telomeres by stabilizing telomeric G4 structures, showing that small molecules can block telomere extension without directly inhibiting the catalytic activity of telomerase [76]. This mechanism can trigger telomere damage signaling more rapidly than the delayed telomere shortening after direct enzymatic inhibition. RHPS4 triggered telomere dysfunction-induced foci (TIFs), which are DNA damage foci formed at dysfunctional telomeres. These foci contained γ-H2AX, phosphorylated RAD17, and 53BP1, indicating activation of a telomeric DNA damage response. Their formation depended on ATR kinase [77]. RHPS4 also displaced POT1 from the single-stranded 3′ telomeric overhang. POT1 normally protects the overhang and limits ATR-dependent damage signaling [11]. By contrast, TRF2 remained associated with telomeres during the early response. TRF2 loss and reduction in the 3′ overhang occurred only after prolonged treatment, indicating later and broader telomere uncapping [77]. BRACO-19 also interfered with telomerase function and telomere capping, leading to telomere shortening and chromosomal abnormalities [78].

Some G4 ligands can disrupt telomere replication at more than one step. Telomere completion does not end with G-strand extension by telomerase. After telomerase extends the G strand, the complementary C strand must also be synthesized to complete telomere end replication. This C-strand fill-in step depends on the CTC1-STN1-TEN1–polymerase α–primase (CST–Polα-primase) complex, which binds telomeric single-stranded DNA [105]. Small-molecule G4 ligands, including TMPyP4 and pyridostatin, inhibited telomeric C-strand synthesis by preventing CST–Polα-primase from binding telomeric single-stranded DNA [79].

G4 stabilization interferes with telomerase function indirectly. G4 ligands do not block the catalytic core of telomerase. Instead, they alter the telomeric substrate, limit telomerase access to the 3′ overhang, and can impair C-strand fill-in [76,79]. In some models, G4 stabilization also weakens telomere protection and activates telomere-associated DNA damage signaling [77,78].

Clinical translation of classical telomeric G4 ligands remains limited by selectivity, pharmacological exposure, and toxicity concerns. Telomeric G4 DNA is only one part of the genomic G4 landscape, so G4 ligand activity cannot always be assigned to selective telomerase substrate blockade. Pyridostatin, for example, induced replication- and transcription-dependent DNA damage at multiple G4-enriched gene bodies [106]. RHPS4 showed effects on normal neural and endothelial cells and additional off-target liabilities in preclinical models [107]. BRACO-19 showed poor permeability in epithelial cell models despite good aqueous solubility [108]. It was also unstable under physiological conditions, and its degradation products showed lower telomerase-inhibitory activity [109]. More recently, BRACO-19 was shown to affect the differentiation of normal human mesenchymal stem cells even at non-cytotoxic concentrations, indicating that its effects are not restricted to cancer cells [110]. These limitations may help explain why classical telomeric G4 ligands remain mainly preclinical despite their strong mechanistic activity.

TMPyP4 is relevant here because it can act in different experimental contexts. Without photoactivation, it can stabilize telomeric G4 structures and may reduce hTERT expression through G4 motifs in the hTERT promoter [75,79,80]. Under photodynamic therapy (PDT) conditions, TMPyP4 can also act as a photosensitizer and generate ROS after light activation [111,112]. Section 5.5 discusses TMPyP4-PDT separately.

### 4.3. Repression of hTERT Expression

Repressing *hTERT* expression lowers telomerase output before the enzyme can engage chromosome ends [113]. hTERT is the catalytic and rate-limiting subunit of telomerase, whereas hTERC is more broadly expressed [114]. Reducing *hTERT* transcription decreases the cellular pool of active telomerase [113].

G4 stabilization can also repress *hTERT* expression at the promoter level. The *hTERT* core promoter contains a G-rich region that folds into stacked G4 conformations [75,115]. TMPyP4, a cationic porphyrinic G4 ligand, was also shown to downregulate c-MYC and reduce *hTERT* expression in tumor models [80]. This promoter-level effect differs from telomeric substrate blockade because it reduces hTERT availability rather than directly altering the telomeric 3′ overhang.

Cells can also suppress *hTERT* transcription through a repressive chromatin state [81]. Class I histone deacetylases (HDACs) remove acetyl groups from histones, promote chromatin compaction, and reduce access of the transcriptional machinery to the *hTERT* locus. HDAC1/2-containing corepressor complexes, including SIN3 transcription regulator family member B (Sin3B), nucleosome remodeling and deacetylase (NuRD), and corepressor of RE1-silencing transcription factor (CoREST), help maintain the *hTERT* promoter in a transcriptionally inactive state [81]. This chromatin state keeps *hTERT* transcription low and limits the amount of active telomerase.

DNA methylation also regulates *hTERT* transcription, but promoter methylation does not simply silence *hTERT* expression. The *TERT* hypermethylated oncological region (THOR) lies upstream of the core *TERT* promoter, and its methylation status influences promoter activity [116]. Unmethylated THOR represses promoter activity, whereas THOR hypermethylation weakens this repression and helps maintain *TERT* transcription [82,116]. Methylation in this region appears to relieve an upstream repressive element rather than silence the promoter itself. In breast cancer, THOR is hypermethylated in malignant tissue, which correlates positively with *hTERT* mRNA expression, and distinguishes malignant from benign tissue in early disease stages [117]. Similar associations in other tumor types indicate that THOR regulation is not restricted to breast cancer [82,118].

### 4.4. Oligonucleotide Inhibitors and Telomerase-Dependent Nucleoside Analogs

Oligonucleotide-based direct telomerase inhibitors can act as competitive inhibitors by binding to the template region of the telomerase RNA component, hTERC. Imetelstat, also known as GRN163L, is a highly potent lipid-conjugated oligonucleotide that binds this region and prevents telomerase from using its RNA template to add telomeric repeats to chromosome ends. In cancer cells, this mechanism reduces telomere maintenance and may lead to progressive telomere shortening during continued proliferation [73,74]. Imetelstat targets the canonical function of telomerase rather than telomeric DNA structure itself.

6-thio-2′-deoxyguanosine (6-thio-dG) acts through a different telomerase-dependent mechanism. After intracellular conversion to its triphosphate form, 6-thio-dG can be incorporated by telomerase into newly synthesized telomeric DNA. This produces modified telomeric repeats and induces telomere dysfunction in telomerase-positive cells [84]. A recent mechanistic study showed that, after incorporation into telomeric repeats, 6-thio-dG inhibits further telomerase-mediated telomeric DNA synthesis by inducing a non-productive, stalled telomerase complex. This effect was more pronounced in cells with short telomeres, linking 6-thio-dG sensitivity to telomere reserve [83]. Unlike imetelstat, 6-thio-dG induces telomeric stress after telomerase incorporates the modified nucleotide into newly synthesized telomeric DNA.

Imetelstat is the most clinically advanced telomerase-targeting agent. It received approval by the United States Food and Drug Administration (FDA) in 2024 and was authorized in the European Union in 2025 as Rytelo following assessment by the European Medicines Agency (EMA) for selected patients with lower-risk myelodysplastic syndromes and transfusion-dependent anemia [119,120]. Direct small-molecule inhibitors, most G4 ligands, and telomerase-dependent nucleoside analogs remain mainly preclinical or investigational in oncology.

## 5. Telomerase- and Telomere-Associated Anticancer Strategies—Translational Aspect

### 5.1. Targeting Telomerase and Treatment Response

Treatment response depends not only on the primary drug target, but also on how cancer cells tolerate therapy-induced stress. Telomerase can influence this tolerance through telomere-dependent and telomere-independent mechanisms. The canonical, telomere-dependent mechanism becomes most relevant when telomeres are short, dysfunctional, or further damaged by treatment. Telomerase maintains or restores chromosome end protection and can limit persistent DNA damage signaling at telomeres [86,87,121]. The non-canonical, telomere-independent mechanism may occur before measurable telomere shortening, because hTERT can affect mitochondrial function, redox balance, apoptotic signaling, and stress adaptation [68,122,123,124,125]. These effects may contribute to treatment resistance when telomere length does not explain the cellular response [68,126].

### 5.2. Targeting Non-Canonical Functions of hTERT

Targeting telomerase may sensitize cancer cells to anticancer treatments by reducing their capacity to tolerate treatment-induced genotoxic, oxidative, or mitochondrial stress [126,127,128].

Acute hTERT depletion sensitized carcinoma cells to DNA-damaging agents and increased their susceptibility to oxidative stress, but did not increase sensitivity to apoptosis induced through the CD95/Fas death receptor, which activates the extrinsic apoptotic pathway. These effects did not require p53 but depended on BAX, a pro-apoptotic Bcl-2 family protein that promotes mitochondrial outer membrane permeabilization [126]. Thus, hTERT depletion enhanced the response to genotoxic treatment and increased susceptibility to oxidative stress through a BAX-dependent mitochondrial apoptotic pathway before telomere shortening became relevant.

In breast cancer models, genetic inhibition of hTERT increased sensitivity to chemotherapy. Silencing hTERT with small interfering RNA (siRNA) reduced hTERT expression, decreased telomerase activity, and enhanced apoptosis after low-dose doxorubicin. The effect on apoptosis was additive, indicating potentiation of doxorubicin cytotoxicity rather than synergy [127]. A dominant-negative hTERT mutant, a catalytically inactive form that interferes with endogenous telomerase function, also sensitized breast tumor cells to doxorubicin and paclitaxel [128]. In this model, telomerase inhibition reduced telomerase activity, induced telomere shortening, slowed cell growth, and increased senescence or apoptosis depending on the drug. Telomerase activity was later detected in treatment-resistant clones, indicating that telomerase suppression was not maintained during long-term selection [128].

Stable hTERT downregulation in breast cancer cells increased doxorubicin sensitivity and reduced clonogenic survival, as shown by decreased colony formation after treatment. It also impaired focal adhesion kinase (FAK)-mediated adhesion, reduced migration, altered β1-integrin- and FAK-driven pathways, and induced autophagy-related changes [129].

Pharmacological telomerase inhibition has also been tested as a chemosensitizing strategy, but the response was model-dependent. TMPyP4 and BIBR1532 were combined with cisplatin, doxorubicin, or paclitaxel in cancer cell lines with different basal hTERT levels and in a non-tumorigenic control line [130]. Drug interactions were assessed formally using the Chou–Talalay method, so synergy was not inferred from increased cytotoxicity alone.

As demonstrated, telomerase inhibition sensitizes only selected telomerase-positive models to chemotherapy. Long-term BIBR1532 treatment shortened telomeres in germ cell tumor cells, but did not increase cisplatin sensitivity or accelerate telomere shortening when combined with cisplatin [131]. The response to telomerase inhibition, therefore, depends on telomere reserve, biological context, and the drug used in combination.

In the treatment-response context, 6-thio-dG provides an additional example of telomerase-dependent sensitization. In telomerase-positive neuroblastoma models, 6-thio-dG enhanced the effects of etoposide or doxorubicin, although the response remained model-dependent [132]. These examples show that sensitization is not a uniform consequence of telomerase inhibition, but depends on inhibitor class, telomere reserve, cellular background, and the co-administered therapeutic agent. As discussed in Section 2.3, alternative TERT splicing may provide an additional source of variability in treatment response, because some catalytically inactive variants retain telomere-independent biological functions.

Beyond its canonical role in telomere maintenance, hTERT has also been linked to DNA damage response and repair. Ectopic hTERT expression enhanced DNA repair kinetics before substantial telomere lengthening was detectable [133]. In normal human fibroblasts, hTERT expression increased nucleotide excision repair and DNA end-joining capacity [134]. hTERT suppression impaired the response to DNA double-strand breaks, reduced repair capacity, and increased radiosensitivity without detectable short-term changes in telomere integrity [124]. In transformed ALT cells, TERT expression accelerated double-strand break repair and increased tolerance to DNA-damaging agents even when telomere synthesis was defective [135]. However, *Tert*-deficient murine cells without critically shortened telomeres showed no attenuation of the ataxia telangiectasia mutated (ATM)-dependent DNA damage response to irradiation and etoposide [136]. TERT may therefore affect DNA damage processing in a model-dependent manner rather than act as a general DNA-repair factor.

Selected non-canonical functions of hTERT, together with their proposed mechanisms, representative experimental models, and relation to telomere length, are summarized in Table 3.

### 5.3. hTERT and the Threshold of Oxidative Cell Death

Many anticancer treatments increase oxidative stress or impair mitochondrial function. Cancer cells often operate close to their redox tolerance limit, so additional stress can push them toward cell death [141,142,143]. hTERT may raise this threshold by stabilizing mitochondrial function and limiting ROS-dependent death signaling [68].

hTERT overexpression reduced basal and stress-induced ROS levels, improved mitochondrial function, increased antioxidant capacity, and limited ROS-mediated apoptosis after oxidative challenge [68]. Acute hTERT depletion had the opposite effect: it increased susceptibility to genotoxic and oxidative stress before detectable telomere shortening [126]. This places hTERT in an early, telomere-independent stress response. The contrasting effects of mitochondrial TERT and possible explanations for these differences are discussed in Section 3.4 and Section 3.5.

### 5.4. The Effect of Telomerase Inhibition on the Radiosensitization of Cancer Cells

Ionizing radiation induces DNA strand breaks, oxidative base damage, and complex DNA lesions [144,145]. Cancer cells survive irradiation when they repair these lesions or tolerate their consequences. Telomerase inhibition may increase radiation response by promoting telomere dysfunction or altering DNA damage processing after irradiation [90,146,147].

The effect of hTERT on the response to genotoxic stress depends on baseline telomere status, including telomere length and pre-existing telomere dysfunction. In human fibroblasts, hTERT increased resistance to ionizing radiation only when telomeres were short and close to dysfunction. Cells with long telomeres did not show the same protection. In this model, hTERT improved survival mainly by restoring telomere function rather than by directly preventing irradiation-induced DNA damage [121]. In a breast cancer model, GRN163L/imetelstat induced telomere dysfunction and increased radiation sensitivity both in vitro and in vivo [147]. The stronger radiation response was associated with telomere dysfunction, indicating a telomere-dependent component of radiosensitization. This may be mechanistically relevant because G-rich telomeric DNA is particularly sensitive to oxidative damage, although telomeric 8-oxo-G formation was not directly assessed in this model.

Telomerase inhibition may also alter DNA damage processing after irradiation. Antisense inhibition of hTERC, especially with GRN163L, enhanced cell death after combined hyperthermia and ionizing radiation [146]. Cells showed delayed formation and resolution of phosphorylated histone H2AX (γ-H2AX) foci and reduced chromosome repair. Because γ-H2AX marks DNA double-strand break sites, delayed γ-H2AX kinetics suggested impaired processing of radiation-associated DNA damage, not simply delayed telomere shortening.

In NSCLC models, low, non-toxic concentrations of BIBR1532 enhanced radiosensitivity after irradiation. Co-treatment increased telomere dysfunction, chromosomal instability, apoptosis, senescence, and mitotic catastrophe, and reduced activity of the ATM–checkpoint kinase 1 (CHK1) pathway [90]. ATM and CHK1 are key components of DNA damage checkpoint signaling that delay cell-cycle progression after genotoxic stress and support DNA repair. Reduced ATM–CHK1 activity may weaken checkpoint control after irradiation and allow unrepaired DNA damage to accumulate.

Telomeric G4 targeting may also enhance the response to irradiation. Pt-ctpy, a platinum-derived G4 ligand, increased radiosensitivity in glioblastoma and NSCLC models both in vitro and in vivo [148]. A similar radiosensitizing effect was reported for the telomerase-dependent nucleoside analog 6-thio-dG in NSCLC models, with increased γ-H2AX levels after irradiation [149]. Increased γ-H2AX indicates stronger DNA damage signaling after irradiation.

Telomerase targeting can enhance the response to irradiation through telomere dysfunction, impaired DNA damage processing, or diminished checkpoint control. The mechanism depends on the inhibitor class and cellular context. Without formal interaction analysis, increased radiosensitivity should be described as radiosensitization or potentiation rather than synergy (Box 1).

### 5.5. TMPyP4 as a G4 Ligand and Photosensitizer: Two Distinct Mechanisms of Action

TMPyP4 can affect treatment response through two distinct mechanisms of action: G4-mediated effects on telomere and telomerase function, and PDT-induced oxidative stress. As a G4 ligand, TMPyP4 can stabilize telomeric G4 structures, limit telomerase access, and reduce hTERT expression. In breast cancer models, these effects also extended to altered adhesion, migration, and spheroid formation, suggesting effects beyond direct cytotoxicity [150,151]. Under PDT conditions, its porphyrin structure enables photosensitizing activity and ROS-mediated phototoxicity. In breast cancer tumoroids, TMPyP4-PDT reduced cell viability, but the response depended on tumor cell type, stromal compartment, and oxygenation state [112]. TMPyP4 effects without photoactivation should be interpreted separately from TMPyP4-PDT.

#### 5.5.1. PDT as a Source of ROS

PDT induces acute oxidative stress through the combined action of a photosensitizer (PS), light, and molecular oxygen. After irradiation, the PS reaches an excited state and generates ROS through type I or type II reactions, or both. In type I reactions, the excited PS reacts with nearby substrates by electron or hydrogen atom transfer, producing radical species and secondary oxidants such as O_2_^•−^, H_2_O_2_, and ^•^OH. In type II reactions, the excited PS transfers energy directly to molecular oxygen, generating singlet oxygen (^1^O_2_) [111]. These ROS can damage mitochondria, lipids, proteins, and nucleic acids, and may reduce viability or trigger cell death [152].

Oxygen availability can limit the efficacy of PDT in solid tumors [153]. Type II reactions depend directly on molecular oxygen, so hypoxia can reduce ^1^O_2_ generation and weaken phototoxicity. Type I reactions are less directly oxygen-dependent and may still generate ROS under low oxygen, but this effect depends on the PS and the experimental model. In hypoxic monolayer cultures, PDT activity may remain detectable, whereas hypoxic 3D models, including spheroids, can show a weaker response [111].

Hypoxia can weaken PDT by limiting oxygen-dependent ROS production and activating stress-adaptation pathways. Low oxygen stabilizes hypoxia-inducible factor 1-alpha (HIF-1α), the oxygen-sensitive subunit of hypoxia-inducible factor 1. HIF-1α promotes genes involved in survival, glycolytic metabolism, and angiogenesis, including vascular endothelial growth factor (VEGF) [154]. In tumor models, hTERT also increased VEGF expression by interacting with specificity protein 1 (Sp1), independently of telomere elongation [140]. Under hypoxic PDT conditions, *HMOX1* expression increased while phototoxicity decreased [111]. *HMOX1* encodes heme oxygenase-1 (HO-1), an NRF2-regulated antioxidant enzyme. HO-1 degrades heme and can reduce ROS-mediated cellular damage [155]. In ovarian cancer models, H_2_O_2_ enhanced TMPyP4-PDT cytotoxicity and increased ROS production, mitochondrial dysfunction, and DNA damage. It also reduced HIF-1α signaling under hypoxia [156].

#### 5.5.2. TMPyP4 as a G4 Ligand and Potential Photosensitizer

The mitochondrial and redox effects of hTERT discussed in Section 3.4 and Section 5.3 may also influence cellular responses to PDT-induced oxidative stress.

Light exposure strongly increased TMPyP4 toxicity, with oxidative stress contributing more than G4 binding alone [157]. Light-activated TMPyP4 titanium dioxide (TiO_2_) nanoparticles generated ROS and reduced cancer cell viability more strongly than free TMPyP4 or TiO_2_ nanoparticles alone [158].

TMPyP4 activity also depends on dose and redox context. Low TMPyP4 concentrations increased cell adhesion and migration, whereas higher concentrations inhibited proliferation and induced cell death [159]. Under H_2_O_2_-induced oxidative stress, TMPyP4 increased ROS production, mitochondrial dysfunction, DNA damage, and apoptosis [160]. Although this was not a PDT model, additional oxidative pressure increased the mitochondrial and DNA damage components of TMPyP4 cytotoxicity. Cells also showed a higher BAX/Bcl-2 ratio and caspase-3 activation, consistent with mitochondrial apoptosis [160].

In breast cancer models, TMPyP4 reduced hTERT mRNA, telomerase activity, and hTERT protein levels, but did not primarily induce rapid apoptosis. Instead, TMPyP4 reduced adhesion and migration, with lower levels of β1-integrin, focal adhesion kinase (FAK), and paxillin [150]. Together, these proteins regulate binding to the extracellular matrix, focal adhesion signaling, and cell migration [161].

#### 5.5.3. Alternative Cell Death Pathways and Immune Signaling

PDT can change the mode of cell death depending on photosensitizer localization, ROS level, and damage intensity. Besides apoptosis, necrosis, and autophagy, PDT may induce several non-conventional death pathways, including mitotic catastrophe, necroptosis, ferroptosis, pyroptosis, paraptosis, and parthanatos. This is relevant to resistant cells, which may limit apoptosis but remain vulnerable to checkpoint failure, lipid peroxidation, regulated necrosis, or immunogenic cell death (ICD) [152].

TMPyP4 may add G4-related genotoxic stress to ROS-mediated damage. As demonstrated, it induced DNA damage and G2/M arrest in a colorectal cancer model [162]. The G2/M checkpoint prevents cells with damaged DNA from entering mitosis. Persistent checkpoint activation or defective checkpoint control can lead to mitotic failure, connecting G4-induced DNA damage with mitotic catastrophe. TMPyP4 also activated the cGAS-STING pathway in this G4-stabilization model [162]. Cyclic GMP-AMP synthase (cGAS) detects cytosolic DNA and activates stimulator of interferon genes (STING), which induces type I interferon and inflammatory signaling [163].

In ICD, dying cells expose calreticulin on the cell surface and release ATP and HMGB1. These damage-associated molecular patterns (DAMPs) support the recruitment and activation of antigen-presenting cells and can promote antitumor immune responses [152]. In TMPyP4-PDT, G4-related stress and ROS-mediated damage may together increase cytotoxic pressure. PDT can engage ferroptosis and ICD, whereas TMPyP4-induced G4 stress can activate cGAS-STING signaling [152,162].

TMPyP4-PDT represents a combined stress-inducing approach rather than proven synergy. TMPyP4 can affect G4-dependent DNA structures, telomerase-related processes, adhesion, and mitochondrial apoptotic signaling, whereas PDT adds acute ROS-mediated damage. Together, these effects may increase cytotoxic pressure and reduce cancer cell tolerance to oxidative and genotoxic stress.

## 6. Summary and Future Perspectives

Telomerase remains a relevant therapeutic target because many cancer cells depend on it for long-term proliferation. Its role, however, is not limited to telomere elongation. hTERT is also linked to cellular stress responses, mitochondrial homeostasis, redox regulation, apoptosis control, DNA damage signaling, and treatment tolerance [123,125,164]. These functions may affect how cancer cells respond to oxidative, genotoxic, mitochondrial, and therapy-induced stress.

Telomerase targeting should therefore not be understood only as delayed telomere shortening. Direct catalytic inhibition mainly affects long-term telomere maintenance and usually requires progressive telomere attrition before strong cytotoxic effects appear [69,70,72]. Other approaches may act earlier. hTERT downregulation, G4 stabilization, mitochondrial hTERT modulation, and telomerase-dependent nucleoside analogs can affect stress-response pathways before measurable telomere shortening [51,68,77,79,84,88]. The biological effect depends on the inhibitor class, telomere reserve, basal hTERT level, redox state, mitochondrial function, and DNA damage response capacity.

The therapeutic effect of telomerase inhibition is context-dependent. In some models, inhibition of telomerase or hTERT sensitizes cancer cells to chemotherapy, irradiation, or PDT-related damage and increases susceptibility to oxidative stress [90,112,126,129,132]. In other models, the effect is weak or absent [131]. Telomerase inhibition should therefore not be treated as a single uniform anticancer strategy.

Clinical use of telomerase inhibitors remains limited. Imetelstat is the only approved telomerase inhibitor and is approved for a defined group of patients with lower-risk myelodysplastic syndromes and transfusion-dependent anemia [119,120]. This shows that telomerase inhibition can provide clinical benefit, but mainly in a selected hematologic disease. The same approach has not yet worked as a broader anticancer strategy. In a randomized phase II trial in advanced NSCLC, maintenance imetelstat did not improve progression-free survival and increased hematologic toxicity [165]. These data suggest that telomerase inhibition is feasible in patients, but its benefit depends on disease context, toxicity, and patient selection.

Several factors may explain why telomerase-targeted therapies have not become broadly effective cancer treatments. Canonical telomerase inhibition often requires repeated cell divisions before telomere shortening becomes biologically limiting. This delayed effect may be too slow for rapidly progressive tumors. Toxicity is another limitation, because selected normal proliferative compartments also express telomerase. This can contribute to hematologic toxicity and limit dose intensity. Patient selection is also difficult. Telomere length, telomerase activity, hTERT expression, ALT status, and non-canonical hTERT functions do not measure the same biological state. Selection based only on tumor type may therefore group tumors with very different dependence on telomerase. Many preclinical agents, including direct small-molecule inhibitors and classical G4 ligands, still lack a clear therapeutic window because of pharmacokinetic limitations, incomplete selectivity, or toxicity in non-malignant cells.

Future studies should not treat telomerase inhibition as one broad strategy for all telomerase-positive tumors. They should first define which tumors depend on canonical telomere maintenance and which depend on early non-canonical hTERT functions. This will require biomarkers that separate telomere length, telomerase activity, hTERT expression, ALT status, and stress-response phenotypes. Studies should also assess drug exposure, toxicity, and treatment combinations in models that more closely reflect the tumor microenvironment, including 3D cultures, co-culture systems, hypoxia, and in vivo models. In this context, telomerase targeting may be more useful as a selected sensitization strategy than as a universal anticancer monotherapy.

## Figures and Tables

**Figure 1:**
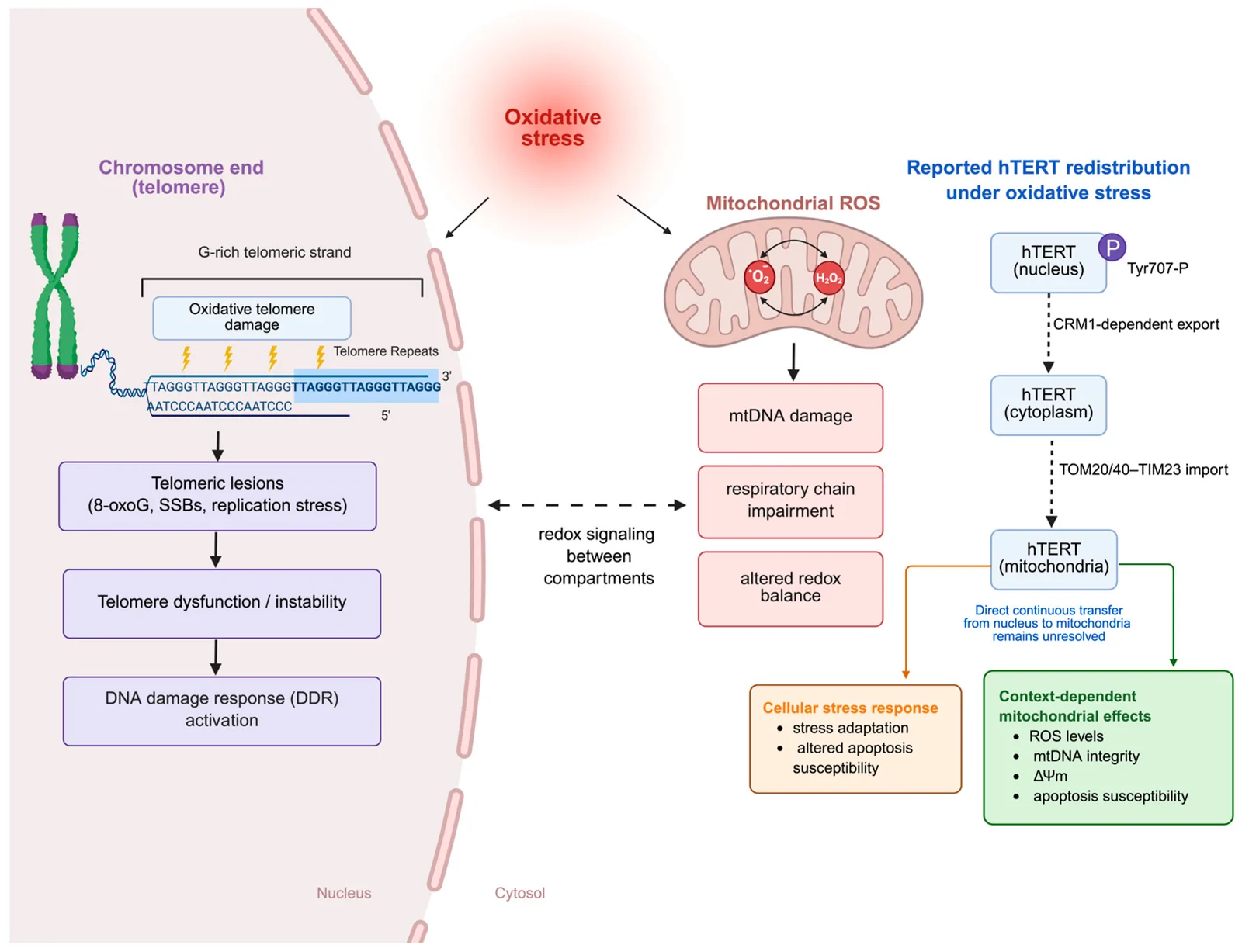
Proposed links between oxidative stress, telomere damage, mitochondrial dysfunction, and hTERT redistribution. Oxidative stress can induce lesions within guanine-rich telomeric repeats, including 8-oxo-7,8-dihydroguanine (8-oxoG), single-strand breaks (SSBs), and replication-associated stress. These lesions may promote telomere dysfunction and activation of the DNA damage response (DDR). Mitochondria are both sources and targets of reactive oxygen species (ROS). Mitochondrial ROS can damage mitochondrial DNA (mtDNA), impair respiratory chain function, and alter redox balance, supporting redox signaling between cellular compartments. Under oxidative stress, human telomerase reverse transcriptase (hTERT) has been reported to redistribute or undergo extranuclear localization. Nuclear export has been linked to Tyr707 phosphorylation and chromosome region maintenance 1 (CRM1)-dependent export, whereas mitochondrial import may involve translocases of the outer mitochondrial membrane 20 and 40 (TOM20/40) and translocase of the inner mitochondrial membrane 23 (TIM23). Direct continuous transfer of the same hTERT molecule from the nucleus to mitochondria remains unresolved. Mitochondrial hTERT may modulate ROS levels, mtDNA integrity, mitochondrial membrane potential (ΔΨm), and apoptosis threshold in a context-dependent manner. These processes may influence cellular stress adaptation and susceptibility to apoptosis. Created in BioRender. Rubis, B. (2026) https://BioRender.com/hgaa6yr (accessed on 8 July 2026).

**Table 1:** Functional specialization of the shelterin complex components (based on [10]).

Protein	Binding Site/Interaction	Role of Telomeres
TRF1 (telomere repeat binding factor)	dsDNA telomeric repeats (TTAGGG)_n_	Binds telomeric dsDNA and contributes to telomere length homeostasis and efficient telomere replication.
TRF2 (telomere repeat binding factor 2)	dsDNA telomeric repeats (TTAGGG)_n_	Stabilizes protective higher-order telomeric structures (T-loop) and suppresses inappropriate DNA damage signaling and end-joining at chromosome termini, preventing telomere fusions.
POT1 (protection of telomeres)	ssDNA 3′ G-rich overhang; ssDNA–dsDNA junction	Binds telomeric ssDNA and limits binding of generic ssDNA-binding factors, thereby restraining damage signaling at telomeres; contributes to regulation of telomerase action at the overhang.
TIN2 (TRF interacting nuclear)	Bridges TRF1/TRF2 to TPP1–POT1	Central scaffold linking dsDNA- and ssDNA-binding modules, stabilizing shelterin architecture and coordinating telomere protection with length regulation.
TPP1 (POT1 interacting protein)	Interacts with POT1; contacts telomerase	Promotes telomerase recruitment and productive telomere elongation, including enhanced telomerase processivity.
RAP1 (repressor/activator protein)	Interacts with TRF2 (no direct DNA binding)	Modulates telomere protection through TRF2 and helps restrain aberrant repair and recombination at telomeres.

dsDNA, double-stranded DNA; ssDNA, single-stranded DNA; T-loop, telomeric loop.

**Table 2:** Classification of telomerase inhibitors in the context of cellular stress response.

Main Category	Subgroup	Main Target	Representative Examples	Potential Relevance to hTERT and Cellular Stress Response
Direct inhibitors	Direct enzymatic inhibitors	hTERT catalytic activity within the telomerase complex	BIBR1532, MST-312	They reduce telomerase activity and may weaken survival pathways in telomerase positive cells [69,70,71,72]
	Oligonucleotide inhibitors	hTERC template region	Imetelstat	They block telomerase function directly and reduce telomere maintenance [73,74]
Indirect inhibitors	G4 stabilizers	Telomeric DNA or G4 motifs within the hTERT promoter	TMPyP4, RHPS4, BRACO-19	They limit telomerase access to telomeres and may also reduce hTERT expression [75,76,77,78,79,80]
	Repressors of hTERT expression	TERT transcription or signaling pathways regulating telomerase	Curcumin, perifosine, rapamycin	They lower hTERT levels and may reduce the ability of cancer cells to cope with stress [81,82]
	Nucleoside analogs	Telomeric DNA after telomerase dependent incorporation	6-thio-dG, AZT, 5-FdU	They induce telomere dysfunction, activate DNA damage response, and weaken stress tolerance [83,84]

**Table 3:** Selected non-canonical functions of hTERT: proposed mechanisms, relation to telomere length, and experimental evidence.

Non-Canonical hTERT Function	Proposed Mechanism	Relation to Telomere Length	Models/Evidence
Mitochondrial homeostasis and redox regulation	Mitochondrial localization; mtDNA interaction; modulation of ROS, membrane potential, respiration, and apoptosis	Can precede detectable telomere shortening	Fibroblast, endothelial, and cancer models; multiple studies, context-dependent effects [51,52,58,59,60,61,62,63,64,68]
DNA damage response and repair	Chromatin modulation; altered DNA damage processing and repair kinetics	Can occur before measurable telomere length changes	Human fibroblasts and transformed ALT cells; multiple studies, with conflicting murine evidence [124,133,134,135,136]
Wnt/β-catenin signaling	BRG1/β-catenin interaction; association with Wnt target chromatin	Not directly tested; TERC and catalytic activity not required in original models	Human cells, mouse ES cells/tissues, Xenopus; inconsistent replication [137,138]
NF-κB signaling	p65 interaction; recruitment to NF-κB-responsive promoters	Not established	Cancer cells, mice, patient-derived leukemia cells; one mechanistic study [139]
VEGF/Sp1 signaling	Sp1 interaction; increased VEGF transcription and angiogenic activity	Not established	Cancer/endothelial cells, mice, human tumors; one mechanistic study [140]

## Data Availability

No new data were created or analyzed in this study. Data sharing is not applicable to this article.

## Abbreviations

The following abbreviations are used in this manuscript:

TERT	telomerase reverse transcriptase
hTERT	human telomerase reverse transcriptase
DNA	deoxyribonucleic acid
TRF1	telomeric repeat-binding factor 1
TRF2	telomeric repeat-binding factor 2
POT1	protection of telomeres 1
TIN2	TERF1-interacting nuclear factor 2
TPP1	POT1-interacting protein
RAP1	repressor/activator protein 1
dsDNA	double-stranded DNA
T-loop	telomeric loop
ssDNA	single-stranded DNA
D-loop	displacement loop
RNP	ribonucleoprotein
RNA	ribonucleic acid
hTERC	human telomerase RNA component
hTR	human telomerase RNA
DKC1	dyskerin
NOP10	NOP10 ribonucleoprotein
NHP2	NHP2 ribonucleoprotein
GAR1	glycine-arginine-rich protein 1
ALT	alternative lengthening of telomeres
ATRX	ATRX chromatin remodeler
DAXX	death domain-associated protein
GABP	GA-binding protein
ROS	reactive oxygen species
SOD2	manganese superoxide dismutase
H_2_O_2_	hydrogen peroxide
mtDNA	mitochondrial DNA
CRM1	chromosome region maintenance 1
MTS	mitochondrial targeting sequence
TOM20/40	translocases of the outer mitochondrial membrane 20 and 40
TIM23	translocase of the inner mitochondrial membrane 23
NADH	nicotinamide adenine dinucleotide, reduced form
ND1	NADH dehydrogenase subunit 1
ND2	NADH dehydrogenase subunit 2
ND3	NADH:ubiquinone oxidoreductase subunit 3
ND5	NADH:ubiquinone oxidoreductase subunit 5
COX1	cytochrome c oxidase subunit 1
^•^OH	hydroxyl radical
AKT	protein kinase B
TERC	telomerase RNA component
IMS	intermembrane space
RNASET2	ribonuclease T2
TERC-53	53-nucleotide mitochondrial TERC fragment
8-oxoG	8-oxo-7,8-dihydroguanine
SSBs	single-strand breaks
DDR	DNA damage response
ΔΨm	mitochondrial membrane potential
BIBR1532	non-nucleosidic small-molecule telomerase inhibitor
MST-312	synthetic small-molecule telomerase inhibitor
G4	G-quadruplex
TMPyP4	5,10,15,20-tetrakis(N-methyl-4-pyridyl)porphyrin
RHPS4	pentacyclic acridine G-quadruplex ligand
BRACO-19	trisubstituted acridine G-quadruplex ligand
6-thio-dG	6-thio-2′-deoxyguanosine
AZT	azidothymidine
5-FdU	5-fluoro-2′-deoxyuridine
IC_50_	half-maximal inhibitory concentration
SA-β-Gal	senescence-associated β-galactosidase
p53	tumor protein p53
p21	cyclin-dependent kinase inhibitor 1A
NF-κB	nuclear factor kappa B
MYC	MYC proto-oncogene, bHLH transcription factor
NSCLC	non-small cell lung cancer
iPSC	induced pluripotent stem cell
BCL2	B-cell lymphoma 2
KRAS	KRAS proto-oncogene, GTPase
VEGF	vascular endothelial growth factor
HIF1α/HIF-1α	hypoxia-inducible factor 1-alpha
TIFs	telomere dysfunction-induced foci
γ-H2AX	phosphorylated histone H2AX
RAD17	RAD17 checkpoint clamp loader component
53BP1	p53-binding protein 1
ATR	ataxia telangiectasia and Rad3-related kinase
CST	CTC1-STN1-TEN1 complex
Polα	DNA polymerase alpha
PDT	photodynamic therapy
HDACs	histone deacetylases
HDAC1/2	histone deacetylases 1 and 2
Sin3B	SIN3 transcription regulator family member B
NuRD	nucleosome remodeling and deacetylase
RE1	repressor element 1
CoREST	corepressor of RE1-silencing transcription factor
THOR	TERT hypermethylated oncological region
mRNA	messenger RNA
GRN163L	imetelstat; lipid-conjugated oligonucleotide telomerase inhibitor
FDA	Food and Drug Administration
EMA	European Medicines Agency
CD95/Fas	Fas cell surface death receptor
BAX	Bcl-2-associated X protein
Bcl-2	B-cell lymphoma 2
siRNA	small interfering RNA
FAK	focal adhesion kinase
β1-integrin	beta-1 integrin
Wnt	Wingless/Int-1 signaling pathway
β-catenin	beta-catenin
ATM	ataxia telangiectasia mutated
CHK1	checkpoint kinase 1
Pt-ctpy	platinum-derived G-quadruplex ligand
PS	photosensitizer
O_2_^•−^	superoxide anion
^1^O_2_	singlet oxygen
3D	three-dimensional
Sp1	specificity protein 1
*HMOX1*	heme oxygenase 1 gene
HO-1	heme oxygenase-1 protein
NRF2	nuclear factor erythroid 2-related factor 2
TiO_2_	titanium dioxide
ICD	immunogenic cell death
G2/M	gap 2/mitosis
cGAS-STING	cyclic GMP-AMP synthase-stimulator of interferon genes
cGAS	cyclic GMP-AMP synthase
STING	stimulator of interferon genes
ATP	adenosine triphosphate
HMGB1	high mobility group box 1
DAMPs	damage-associated molecular patterns
